# Sixteen years of bathymetry and waves at San Diego beaches

**DOI:** 10.1038/s41597-019-0167-6

**Published:** 2019-08-29

**Authors:** B. C. Ludka, R. T. Guza, W. C. O’Reilly, M. A. Merrifield, R. E. Flick, A. S. Bak, T. Hesser, R. Bucciarelli, C. Olfe, B. Woodward, W. Boyd, K. Smith, M. Okihiro, R. Grenzeback, L. Parry, G. Boyd

**Affiliations:** 1 Scripps Institution of Oceanography, University of California, San Diego, La Jolla, CA 92037 USA; 2 Coastal and Hydraulics Laboratory, US Army Engineer Research and Development Center, 1261 Duck Rd, Duck, NC 27949 USA; 3 Coastal and Hydraulics Laboratory, US Army Engineer Research and Development Center, 3909 Halls Ferry Rd, Vicksburg, MS 39180 USA

**Keywords:** Physical oceanography, Geomorphology

## Abstract

Sustained, quantitative observations of nearshore waves and sand levels are essential for testing beach evolution models, but comprehensive datasets are relatively rare. We document beach profiles and concurrent waves monitored at three southern California beaches during 2001–2016. The beaches include offshore reefs, lagoon mouths, hard substrates, and cobble and sandy (medium-grained) sediments. The data span two energetic El Niño winters and four beach nourishments. Quarterly surveys of 165 total cross-shore transects (all sites) at 100 m alongshore spacing were made from the backbeach to 8 m depth. Monthly surveys of the subaerial beach were obtained at alongshore-oriented transects. The resulting dataset consists of (1) raw sand elevation data, (2) gridded elevations, (3) interpolated elevation maps with error estimates, (4) beach widths, subaerial and total sand volumes, (5) locations of hard substrate and beach nourishments, (6) water levels from a NOAA tide gauge (7) wave conditions from a buoy-driven regional wave model, and (8) time periods and reaches with alongshore uniform bathymetry, suitable for testing 1-dimensional beach profile change models.

## Abstract (summary)

**Table:** 

Design Type(s)	database creation objective • modeling and simulation objective
Measurement Type(s)	bathymetry and waves
Technology Type(s)	Oceanography
Factor Type(s)	geographic location
Sample Characteristic(s)	San Diego • ocean

Machine-accessible metadata file describing the reported data (ISA-Tab format)

## Background & Summary

Sustained quantitative observations of nearshore waves and sand levels are costly and few, yet essential for understanding beach change from natural and anthropogenic forcing, and for testing and improving beach evolution models. Remote sensing techniques are being developed for subaqueous sand level monitoring but accuracy is limited, especially in the inner surfzone^1^. Watercraft equipped with a Global Navigation Satellite System (GNSS) and sonar, driven on shore-perpendicular transects, remains the most reliable, albeit labor intensive subaqueous monitoring technique. Subaerial and wading depth monitoring is far more common. Forty years of monthly subaerial surveys at Narrabeen, Australia are accompanied by only 11 subaqeous surveys irregularly spaced in time^2^. Subaerial survey programs lacking repeat subaqueous surveys include Moruya, Australia^3^, Rhode Island, USA^4^, and many others. The New Jersey Beach Profile Network, spanning about 270 km with 171 transects surveyed bi-annually since 1986, documents the extensive subaerial dune erosion of major hurricanes^5^.

Long-term monitoring of multiple subaqueous beach profiles include: (a) Dutch JARKUS program, approximately annual surveys since the 1960s, spanning 115 km with ~250 m alongshore resolution^6^; (b) Columbia River Littoral Cell annual monitoring since 1997, spanning ~165 km of U.S. Northwest Pacific Coast with alongshore resolution between 200 m–1 km^7^; and (c) US Army Corps of Engineers fortnightly monitoring since 1981 of ~1 km of North Carolina coast at ~50 m resolution^8,9^.

**Figure 1:**
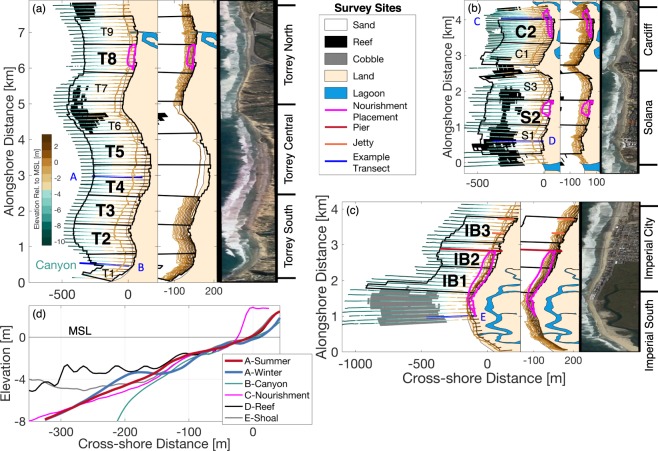
Survey Sites. Overview of bottom elevation observations at (**a**) Torrey Pines, (**b**) Solana-Cardiff, (**c**) Imperial Beach. A 450 m-long, recreational pier (section IB2), and two short 100–150 m jetties (IB3) are indicated. In (**a**–**c**), the left most panel shows quarterly cross-shore transects (colored by depth), bottom type and location of nourishments (see legend). Bold section labels (e.g. T2, T3) indicate alongshore uniformity. Middle panels shows tracks of a typical monthly subaerial survey. Right panels are aerial photographs, with aspect ratio stretched to match the left and center panels. (**d**) Mapped profiles (elevation versus cross-shore distance) for selected transects (blue lines labeled A–E in (**a**–**c**)).

**Figure 2:**
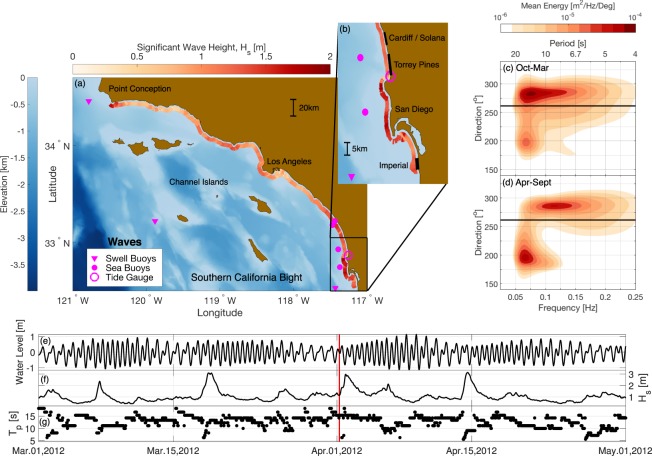
Waves. (**a**) Map of Southern California Bight; offshore swell and local sea buoys, and NOAA tide gauge are indicated (magenta symbols, see legend). Red shaded colors near the shoreline are significant wave height in 10 m depth on April 1, 2012, smoothed with a 2 km moving average. The detailed pattern of spatial variation is highly variable, and depends on the mix of wave directions and periods. This example case illustrates the substantial spatial variation of wave conditions typical of this region under all conditions. (**b**) Inset with study beach locations. (**c**) Winter (Oct-Mar) and (**d**) summer (Apr–Sept) climatological offshore directional spectra at Torrey Pines buoy (northernmost buoy in inset). Black line is average shoreline orientation at study beaches in (**b**). The buoy was used to show general directionality of the region, but note that waves refract and become more shorenormal as they approach the coast. (**e**) Tide elevation relative to MSL, (**f**) wave height and (**g**) peak period versus time at Torrey Pines (10 m depth, location A in Fig. 1a). Red vertical line marks the time of wave conditions in panels (a,b).

Here we describe a dataset of wave estimates and sand level observations collected and curated by the Scripps Institution of Oceanography for three reaches of the San Diego County coastline: *Torrey Pines*, *Cardiff and Solana*, and *Imperial Beach* (Fig. 1 and Table 1). At each site, monitoring spans between 4–8 km alongshore and 8–16 years (2001–2016). The sand level dataset is unique in the large number (165) of closely-spaced (~100 m), quarterly, cross-shore transects from the back beach to 8 m depth. In total, more than 15,000 km of survey track was successfully driven. Similar to refs^2,7^, the narrower, more easily accessible subaerial region was surveyed more often (monthly), using a GNSS-equipped All-Terrain Vehicle (ATV). The study area includes offshore reefs, lagoon and small river mouths, hard substrate, and cobble and sandy sediments. Alongshore variation in waves is caused by wave-shadows of the offshore Channel Islands and refraction over local shelf bathymetry^10^ (Fig. 2a). A Datawell buoy network and regional wave propagation model provide wave estimates in 10 m depth with 100 m alongshore resolution^11^. Water levels are provided from a local National Oceanic and Atmospheric Administration (NOAA) tide gauge (Fig. 2a,e).

**Table 1:** Beaches.

Beach	Alongshore Length [*m*]	MOP lines (inclusive)^a^
Imperial	4200	024–065
Torrey	7900	520–598
Cardiff/Solana	4500	638–682

^a^MOP line names are ‘D0###’ where ### is listed in table.

These datasets have been used to investigate equilibrium behavior of the shoreline^12^ and beach profile^13^, beach response to two energetic El Niño winters^14–19^ and the evolution of four beach nourishments^17,20–23^. The performance of 1-D (imensional) storm erosion models (XBeach)^22^, and 2-D morphological evolution models (Delft3D)^24^ have been assessed. These bathymetry data also have been used in tests of surfzone circulation (funwaveC)^25^, and coastal flood models (XBeach and EurOtop)^26^.

Datasets are provided from 2001, when the sand level monitoring was initiated, through 2016, when a truck-mounted LIDAR^27^ and photogrammetric drone^28^ increased the resolution and span of subaerial monitoring. The creation of the datasets within each repository folder is described in the ‘Methods’ section after a short description of the beach sites and monitoring history. The data folders included are (1) water levels from the La Jolla NOAA tide gauge, (2) wave conditions from a buoy-driven regional wave model (3) sand level survey information (4) raw sand elevations (5) binned sand elevations (6) mapped sand elevations, (7) beach characteristics, and (8) analysis code and files used in processing. Beach characteristics include the locations and properties of (a) Monitoring and Prediction lines, (b) regions, (c) sections, (d) piers and jetties, (e) hard substrates, and (f) nourishments, as well as times of nourishment placement and nourishment influence. (g) Beach width and (h) subaerial and total sand volume times series are also provided as characteristics. The repository is described in the ‘Data Records’ section, and the final sections discuss the details of ‘Technical Validation’ and ‘Usage Notes’.

## Methods

### Site description

The wave climate for the three study sites, (and for San Diego County) exhibits strong seasonal change in both wave energy and direction. In general, the energetic winter waves of proximate north Pacific storms (Fig. 2c) transport beach sand seaward and southward, and milder summer swell from distant south Pacific storms (Fig. 2d) transport sand shoreward and northward. However, island wave blocking, offshore shoals, nearshore submarine canyons, rocky headlands, and relic sediment fans seaward of numerous coastal lagoons all contribute to significant alongshore wave variability (Fig. 2a,b).

Sand at all three sites is medium grained (median *D*_50_ = 0.2 mm) but with considerable alongshore and cross-shore variation (~±0.1 mm)^29,30^. Cobbles are exposed intermittently on the subaerial beaches, especially when sand levels are eroded^31^. Some beaches are backed by seacliffs, generally composed of two geologic units: a bottom unit of lithified Eocene and Miocene mudstone, shale, sandstone, and siltstone, and a top unit of unlithified Pleistocene terrace deposits^32^.

*Torrey Pines* is predominantly backed by cliffs (~50–100 m tall); an exception is a short rip-rap section where the heavily trafficked coastal highway abuts the beach (North Torrrey Pines, Fig. 1a). North Torrey Pines Beach has reef seaward and northward of the Los Peñasquitos lagoon mouth (Fig. 1a, north of section T9) and in section T7. Central Torrey Pines Beach has reef in T6. South Torrey Pines Beach contains the landward tip of the Scripps Submarine canyon (Fig. 1a, lower left corner). Waves and sand level changes at Scripps Canyon and Torrey Pines have been studied predating the present surveys (refs^33–38^ and others).

The longest sand level time series in the study began in 2001 at North Torrey Pines (Table 2) to monitor the evolution of a subaerial beach nourishment (Table 3) placed to protect an adjacent major thoroughfare. A few months after placement, subaerial nourishment sand, constructed with a grain size similar to native, was eroded completely by a moderate storm^20^. Sand was stored in an offshore bar and partially returned to the subaerial beach the following summer^21^. In autumn 2003, waves, currents, and morphology near Scripps Canyon and at South Torrey Pines Beach were observed during the Nearshore Canyon Experiment (NCEX) (refs^24,39,40^, and references therein). In 2004, monitoring expanded to span 8 km alongshore, including both North, Central, and South Torrey Pines (Fig. 1a).

**Table 2:** Regions.

Region	Start Date	Alongshore Length [*m*]	MOP lines (inclusive)^a^	Un-nourished Times Start^b^	Un-nourished Times Finish^b^	Mean (Std) Beach Width [*m*]^c^
Imperial South	14 Nov 2008	1700	024–040	14 Nov 2008	6 Sep 2012	39 (9)
Imperial City	14 Nov 2008	2500	041–065	14 Nov 2008	6 Sep 2012	48 (9)
Torrey South	20 Aug 2003	2400	520–543	20 Aug 2003	31 Dec 2016	60 (10)
Torrey Central	3 Apr 2004	2700	544–570	3 Apr 2004	31 Dec 2016	61 (8)
Torrey North	27 Feb 2001	2800	571–598	6 Feb 2004	31 Dec 2016	39 (8)
Solana	10 Apr 2008	2700	638–664	10 Apr 2008	3 Nov 2012	24 (5)
Cardiff	31 May 2007	1800	665–682	31 May 2007	24 Oct 2012	39 (8)

^a^MOP line names are ‘D0###’ where ### is listed in table.

^b^Un-nourished times are times with minimal nourishment influence.

^c^Mean (and standard deviation) of beach width for times with minimal nourishment influence.

**Table 3:** Nourishments.

Beach	Native Grain Size [*mm*]^a^	Nourishment Grain Size [*mm*]^b^	Nourishment Volume [*m*^3^]^c^	Placement Start	Placement Finish
Torrey North	0.23	0.2	187,000	6 Apr 2001	27 Apr 2001
Imperial	0.25	0.53	344,000	7 Sep 2012	4 Oct 2012
Cardiff	0.16	0.57	68,000	25 Oct 2012	28 Oct 2012
Solana	0.15	0.55	107,000	4 Nov 2012	27 Nov 2012

^a^*D*_50_ at MSL. Torrey, Imperial and Cardiff from ref.^13^. Solana from ref.^56^.

^b^*D*_50_. Torrey from ref.^20^. Imperial, Cardiff, and Solana from ref.^57^.

^c^Refs^57,58^.

*Cardiff and Solana* Beaches have reef in all sections except C2 (Fig. 1b)^13,23^. The San Dieguito Lagoon mouth is at the southern boundary of Solana, while the San Elijo Lagoon mouth borders Cardiff in the north. Cardiff is backed by rip-rap, parking lots and the heavily trafficked coastal highway, whereas Solana is backed by partially sea-walled cliffs (~20–30 m tall).

Solana and Cardiff beaches suffer chronic erosion and occasional flooding of parking lots and park facilities. In extreme cases the highway floods, interrupting traffic and commerce. Cardiff monitoring began in 2007, and in 2008 expanded south to Solana Beach (Fig. 1b and Table 2). Cardiff and Solana were nourished with relatively coarse-grained sand in autumn 2012 (Table 3). Subaerial sand levels remained unnaturally elevated for several years, including periods of energetic waves^17,23^.

*Imperial Beach* contains a large cobble shoal in the south, offshore of the Tijuana River mouth (Fig. 1c). A 450 m-long recreational pier and two short (100–150 m) jetties, are located to the north. Some shoreline is backed by rip-rap and homes, with small dunes elsewhere.

Surveying began in 2009 (Fig. 1c and Table 2), however, due to often polluted estuary discharge, only subaerial measurements are made near the river mouth, and subaqueous surveys of the entire reach are less often than quarterly. Surveys were more frequent and spatially irregular during the autumn IB09 experiment (tracer dye was tracked)^25,41^. In autumn 2012, relatively coarse nourishment sand was placed on the beach (Table 3) and largely remained subaerial for several years, similar to the nourishments at Cardiff and Solana^17^. After a few years of seasonally reversing alongshore drift, the nourishment contributed to clogging the Tijuana River mouth, degrading estuary water quality^23^.

### Water levels

Hourly observed and six-minute predicted water levels at the end of Scripps Pier (average water column depth ~6 m) in La Jolla (magenta circle, Fig. 2a,b) are extracted from the National Oceanic and Atmospheric Administration’s (NOAA’s) Center for Operational Oceanographic Products and Services (CO-OPS) database (https://tidesandcurrents.noaa.gov/stationhome.html?id=9410230). During the extracted record, an acoustic sensor was used until 2014 (Aquatrak air acoustic sensor), when it was fully replaced by a microwave radar water level sensor (Xylem WaterLOG H-3611i, first installed in 2013). A pressure sensor (GE Druck PDCR 4010) was used as a backup to fill in data gaps^42^.

### Waves

To facilitate modeling of beach profile change, wave characteristics in 10 m depth spaced 100 m alongshore were extracted from the Scripps Institution of Oceanography’s wave Monitoring and Prediction (MOP) system for the California coastline^11^, (http://cdip.ucsd.edu/). Winter swell is from Gulf of Alaska storms to the north, whereas summer swell is from the southern Hemisphere^10^, (Fig. 2c-d). Wave estimates along the coastline are produced using a linear spectral refraction model initialized with 2-D spectral estimates from multiple Datawell directional buoys. For swell waves (0.04–0.08 Hz) the model is initialized with deep water buoys located seaward of the Channel Islands (Fig. 2a). For sea waves (0.09–0.5 Hz) the model is initialized with buoys located inside the islands along the mainland shelf break (Fig. 2b). Each MOP point in 10 m depth has a corresponding backbeach point, defining a MOP line. MOP line orientations are chosen to minimize the distance from the backbeach to the 10 m depth contour.

Hindcast time series of wave height (*H*_*s*_), peak (*T*_*p*_) and average (*T*_*a*_) wave period, peak (*D*_*p*_) and mean (*D*_*m*_) wave direction, and radiation stress estimates (onshore *S*_*xx*_ and alongshore *S*_*xy*_) relative to the MOP estimated shore normal (orientation also provided), are provided at the seaward end of each MOP line on the 10 m depth contour. Additionally, time series of wave energy, *E*, and low-order moment directional Fourier coefficients (*a*_1_, *b*_1_, *a*_2_, *b*_2_, in true compass “from” coordinates), as a function of wave frequency, are provided in 10 m depth. The 10 m depth wave model output mirrors the information provided by directional wave buoys^43^ or a pressure-velocity meter (PUV), and can be treated in the same way as the spectral data from these instruments when defining boundary conditions for sediment transport models.

### Survey information

An ATV with rear shocks removed and constant tire pressure (to maintain a consistent distance from the GNSS antenna to the sand level below), was used to measure the subaerial beach at low tide, while a 3 wheeled push dolly (with GNSS antenna mounted on a fixed-height mast) was used from the low-tide waterline to chest deep wading depths. A personal watercraft (Yamaha Waverunner, but here the more familiar term jet ski will be used) equipped with 192 kHz acoustic sonar, sea surface thermistor (for speed of sound calculation) and GNSS antenna, measures the subaqueous profile at high tide. The dolly is used to help ensure data is collected along a continuous profile, through water that is too deep for ATV, and where high turbidity confounds the jet ski sonar. The receivers on the vehicles transitioned from Sokkia, to Ashtech ZXtreme, and are now equipped with Trimble NetR9 GNSS receivers (enabling access to multiple Global Navigation Satellite Systems). The GNSS sample rates have increased over time, and data are now collected at 5 Hz. Base stations broadcast real-time kinematic corrections that allow jet ski and ATV drivers to monitor the data quality, follow designated transect lines, and guide dolly pushers, using custom in-house software. Vehicles are driven at a speed that samples ~1 point per meter of track. Data are routinely post-processed. SBG Ellipse inertial measurement units on the jet ski and ATV account for tilting of the antenna. Prior to the advent of MEMS, a KVH Gyrocompass was used. The ATV driver also manually records subaerial substrate type with a switch that differentiates between rock, cobble and sand.

**Figure 3:**
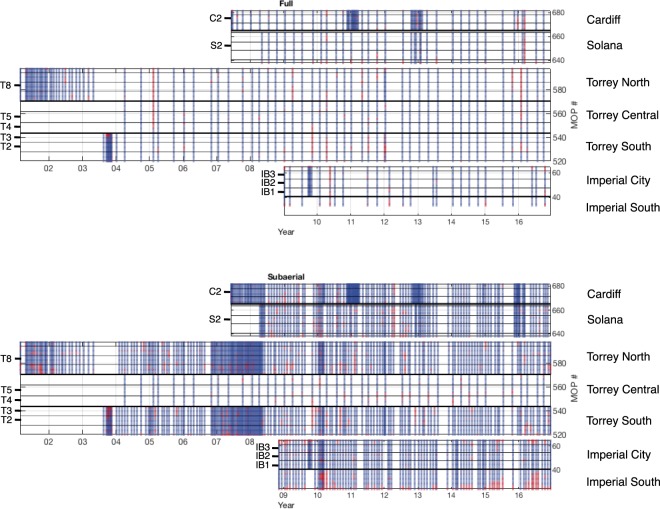
Survey coverage. Survey coverage versus location (MOP number vertical axis) and time (horizontal axis) for (left) subaerial (between the average location of the MSL contour and backbeach) and (right) full (between average location of −6 m contour and backbeach) depth zones. Blue indicates accurate mapping. Red delineates MOP lines missing more than 10% of mapped data.

The location, spacing, and orientation of full survey transects evolved organically over time and space. Full survey transects are by design aligned with MOP lines at Solana and Imperial Beach. At Torrey Pines and Cardiff, surveying began before the creation of the MOP model, and cross-shore transects were orthogonal to the approximate orientation of the mean higher high water contour (MHHW = 1.56 m rel. NAVD88) over a few km alongshore. Cardiff and Solana transects aligned with MOP transects starting November 23, 2011. (Torrey transects were aligned to MOPs in 2017.) Subaerial ATV-only surveys are driven alongshore with approximately 10 m cross-shore spacing. Nominally, full surveys are quarterly and subaerial surveys are monthly (Fig. 3). At times, surveys were more frequent at Torrey Pines (e.g. 2001–03, 2007–08) and Cardiff (e.g. Winter 2010–11 and 2012–13). Complete lists of all survey filenames, dates, depth zones surveyed, alongshore regions surveyed, south and north-most surveyed MOP line indices with good coverage, regions influenced by nourishment, and vehicles and transects driven are included.

### Raw sand elevations

Quality controlled elevation data (NAVD88, GEOID99 epoch 2002) are provided for each survey (Fig. 1a–c) at both Lat-Long (NAD83 CORS96, epoch 2002, ellipsoid GRS80) and UTM (Zone 11) coordinates. When available, subaerial substrate type (sand, rock or cobble) is also provided. Raw (quality controlled) sand level data provide maximum user flexibility. Binned and mapped data (below) are more user-convenient for many applications, but sharp edges are blurred. Raw data should be used to examine vertical scarps at the seaward face of nourishments, and steep reef and canyon bathymetry (Fig. 1d).

### Binned sand elevations

Raw sand level observations are binned to coordinates aligned with the wave estimates (MOP lines). MOP lines are separated 100 m alongshore, and oriented from the 10 m depth contour to the backbeach, to approximately follow the curving coastline (see waves and water level, above). Bins centered on MOP lines with 5 m cross-shore resolution are filled with median values, suppressing the effect of outliers. All surveys are binned, including surveys with transects not originally aligned to MOP lines and with alongshore spacing less than 100 m (e.g. 50 m, Cardiff Fig. 1b). The observations are usually smooth over the 50 m maximum distance of alongshore projection and 2.5 m cross-shore projection. However, raw data should be used to define features with shorter scales (i.e., scarps, reef, canyon). In the repository code, binned alongshore resolution can be adjusted by using different binning transects, while cross-shore resolution can be adjusted by redefining the “cres” variable.

### Mapped sand elevations

Elevation maps are created on the same grid as the binned observations, but are smoothed and fill in small data gaps. For each survey, bins containing less than 3 data points are considered unsampled and are discarded. Map boundaries are defined as grid points that are regularly sampled during unnourished quarterly full surveys (must be populated at least 25% of the time as the most frequently full surveyed grid point, during times without nourishment). Grid points with an unnourished average depth greater than 8 m are not mapped because speed of sound errors due to stratification may contaminate jet ski sonar measurements. As described below, when the estimated interpolation (or extrapolation) error is large, the map bin elevation is considered missing and filled with the value −99999.

The unnourished time-averaged spatially smoothed mean depth, 〈*d*〉_*smooth*_, is removed from the binned observation data, *d*,

1 $$d^{\prime} =d-{\left\langle d\right\rangle }_{smooth}.$$

Estimates of 〈*d*〉_*smooth*_ use unnourished full surveys that include all regions from the site (Table 2). At Cardiff/Solana and Imperial Beaches only full surveys before the 2012 nourishment placement were used to calculate the mean. At Torrey Pines, nourishment influence was minimal by the time monitoring had expanded to all three regions. The mean is spatially smoothed using the same Gaussian filter used to create the maps (described below).

The data fluctuation, *d*′, contains both the true signal fluctuation, *s*′, and noise, $\varepsilon$

2 $$d^{\prime} =s^{\prime} +\varepsilon .$$

where $\varepsilon$ = 2 cm is used. Each mapped fluctuation grid point, *m*′, is a linear combination of the observed data fluctuations

3 $$m^{\prime} ={a}^{T}d^{\prime} .$$

where the mean square error (MSE), 〈*e*^2^〉,

4 $$\left\langle {e}^{2}\right\rangle =\left\langle {(m^{\prime} -s^{\prime} )}^{2}\right\rangle $$

5 $$=\,{a}^{T}\left\langle d^{\prime} {d}^{^{\prime} T}\right\rangle a-2\left\langle d^{\prime} s^{\prime} \right\rangle a+\left\langle {s}^{^{\prime} 2}\right\rangle $$

is minimized with gain,

6 $$a={\left\langle d^{\prime} {d}^{^{\prime} T}\right\rangle }^{-1}\left\langle d^{\prime} s^{\prime} \right\rangle .$$

Noise is assumed uncorrelated with the signal and between gridpoints^44^,

7 $$\left\langle d^{\prime} {d}^{^{\prime} T}\right\rangle =\left\langle s^{\prime} {s}^{^{\prime} T}\right\rangle +\left\langle {\varepsilon }^{2}\right\rangle .$$

A simple Gaussian filter (similar to ref.^45^)

8 $$\left\langle s^{\prime} {s}^{^{\prime} T}\right\rangle =\left\langle {s}^{^{\prime} 2}\right\rangle exp(-{(\Delta {\tilde{y}}/{L}_{{\tilde{y}}})}^{2}-{(\Delta \widetilde{x}/{L}_{\widetilde{x}})}^{2}),$$

is used in a coastline following coordinate frame, where ${\tilde{y}}$ is the MOP line and $\widetilde{x}=0$ is the mean unnourished shoreline location (defined as the average position of the intersection of the profile with the mean sea level contour, MSL = 0.77 m NAVD88) on the MOP line (negative values offshore). The cross-shore smoothing scale is ${L}_{\widetilde{x}}$ = 15 m and alongshore smoothing scale is ${L}_{{\tilde{y}}}$ = 2 MOP lines (~200 m). The smoothed mean, 〈*d*〉_*smooth*_, is added to *m*′ to create maps relative to NAVD88. Elevation maps relative to NAVD88, *m*, maps relative to the smoothed mean, *m*′, and the smoothed mean, 〈*d*〉_*smooth*_, are provided.

To test mapping accuracy and error estimates, maps were created with subsampled binned survey data and the interpolation was compared to observed values at the grid points that were decimated to create the subsample. Considerable effort to emulate the spatially complex patterns of the observed autocovariance did not significantly improve results compared with the Gaussian method used here, where smoothing scales were simply chosen to reasonably fill in gaps. The mean square error estimates provided by the mapping technique were found not representative of true errors, however the estimates provide qualitative guidance of the relative distance of grid points from observations.

Normalized MSE (NMSE) at each grid point is estimated as

9 $$NMSE=\left\langle {e}^{2}\right\rangle /\left\langle {s}^{^{\prime} 2}\right\rangle ,$$

where the signal variance is estimated over the entire survey as

10 $$\left\langle {s}^{^{\prime} 2}\right\rangle =\left\langle {d}^{^{\prime} 2}\right\rangle -\left\langle {\varepsilon }^{2}\right\rangle .$$

Mapped data is flagged as missing where NMSE >0.2. This threshold limits excessive extrapolation and also encapsulates that cross-shore gaps larger than about 15 m, caused by suboptimal tide and waves during a survey, create unacceptable uncertainty about sand bar structure. Red (Fig. 3) delineates MOP lines missing more than 10% of mapped data.

### Beach characteristics

#### MOP definitions

The beach site locations are defined using MOP lines (Table 1). Backbeach locations of each line (spaced 100 m apart in the alongshore) and the corresponding offshore locations in 10 m depth are included, as well as the MOP site names, index number, and the angle of the line relative to true north.

#### Regions

The monitoring schemes at each beach evolved over time with consistently surveyed regions spanning between 1.6 and 2.7 km alongshore (Fig. 1a–c and Table 2). Region outlines, and MOP site names and index numbers within each region are provided, as well as times with minimal nourishment influence.

#### Sections

Beaches were split into sections spanning 700–900 m alongshore in the analysis of ref.^13^. For each section, location outlines, MOP sites and index numbers, times of minimal nourishment and other details are provided (Table 4). Sections labeled 1D (bold in Table 4), have a coherent seasonal cross-shore sand exchange signal along the profile, as identified with empirical orthogonal function analysis^13^. During times of minimal nourishment influence, these 1D sections are recommended for testing 1-D cross-shore beach profile evolution models. Note that cobble may be present even in 1D sections, especially at North Torrey Pines and Cardiff when subaerial sand levels are eroded.

**Table 4:** Sections.

Region	Section^a^	Alongshore Length [*m*]	MOP lines (inclusive)^b^	# Sub (Total) Vol^c^	# Sub (Total) Vol Nourished^c^	# Sub (Total) Vol Un-nourished^c^	Features
Imperial City	**IB1**	700	041–047	94 (19)	45 (8)	49 (11)	
	**IB2**	700	048–054	91 (28)	43 (13)	48 (15)	Pier
	**IB3**	700	055–061	92 (26)	42 (12)	50 (14)	Jetties
Torrey South	T1	800	520–527	196 (55)	0 (0)	196 (55)	Canyon
	**T2**	800	528–535	192 (49)	0 (0)	195 (49)	
	**T3**	800	536–543	183 (45)	0 (0)	183 (45)	
Torrey Central	**T4**	900	544–552	42 (41)	0 (0)	42 (41)	
	**T5**	900	553–561	42 (40)	0 (0)	42 (40)	
	T6	900	562–570	42 (41)	0 (0)	42 (41)	Reef
Torrey North	T7	800	571–578	200 (72)	29 (30)	171 (42)	Reef
	**T8**	800	579–586	229 (76)	33 (32)	196 (44)	
	T9	800	587–594	227 (70)	31 (30)	196 (40)	Lagoon
Solana	S1	800	641–648	100 (32)	50 (16)	50 (16)	Reef
	**S2**	700	649–655	100 (34)	47 (16)	53 (18)	Reef
	S3	800	656–663	99 (34)	49 (17)	50 (17)	Reef
Cardiff	C1	600	666–671	181 (57)	63 (21)	118 (36)	Reef
	**C2**	700	672–678	182 (56)	63 (20)	119 (36)	

^a^Alongshore uniform sections in bold^13^.

^b^MOP line names are ‘D0###’ where ### is listed in table.

^c^Number of successful estimates in the subaerial (total) volume time series (examples shown for T8 in Fig. 3b,c). Nourished and Un-nourished correspond to times in Table 2.

#### Pier and jetty locations

The locations of the pier and two short jetties at Imperial Beach are included (Fig. 1c).

#### Nourishment

Sand nourishment placement periods and locations are provided (Table 3). The nourishment placement outline is defined as the bulge in the 2 m (relative to MSL) contour location between the pre- and post-nourishment survey maps (magenta, Fig. 1a–c).

#### Hard substrate

Subaerial substrate is monitored by the ATV driver, however, offshore substrate is difficult to identify. Areas with underlying hard substrate erode to minimum levels significantly less than adjacent sandy areas. Specifically, these areas are defined as areas with mapped *minimum* surface greater than 30 cm relative to the time- and alongshore- *averaged* mapped profile in the alongshore uniform sections (Fig. 1a–c). These locations agree qualitatively with limited available sidescan sonar^23^ which helped to identify the hard substrate at Imperial Beach as a cobble shoal, while the hard substrate at Torrey Pines and Cardiff/Solana is rocky reef.

#### Volumes

**Figure 4:**
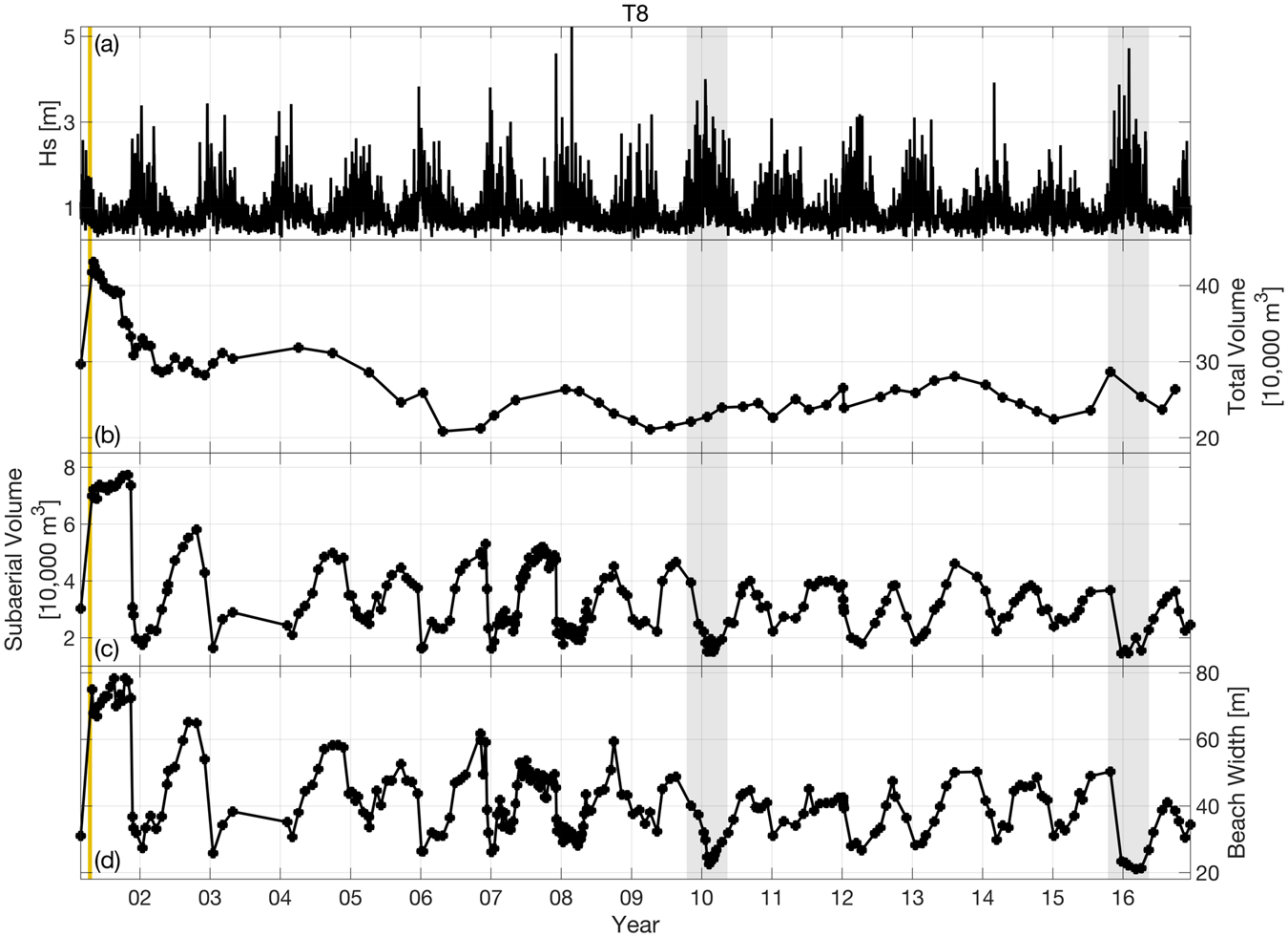
Time series products. An example of time series products (provided for all beaches, regions and sections) is shown for Torrey Pines section T8 (Fig. 1 and Table 4), alongshore averaged over 8 adjacent MOP lines. (**a**) Wave height in 10-m depth, (**b**) total volume, (**c**) subaerial volume, and (**d**) beach width, all versus time. Beach nourishment placement (vertical gold bar, 2001) and El Niño winters (grey bars, 2009/10 and 2015/16) are indicated.

For each survey, maps are used to estimate sand volumes relative to the minimum surface (Fig. 4b,c). The minimum value in each mapped grid point over the study period is used to calculate the minimum surface *h*_*min*_(*x*, *y*). The total volume is estimated over the survey area, *A*_*tot*_,

11 $${V}_{tot}(t)=\mathop{\int }\limits_{{A}_{tot}}[h(x,y,t)-{h}_{min}(x,y)]da$$

while subaerial volume,

12 $${V}_{sub}(t)=\mathop{\int }\limits_{{A}_{sub}}[h(x,y,t)-{h}_{min}(x,y)]da$$

is calculated over *A*_*sub*_ which extends from the mean shoreline position (time-average location of the intersection of the unnourished profile with MSL) to the backbeach. Estimates are provided for each beach (Table 1), region (Table 2) and section (Table 4). Volume estimates are discarded if more than 10% of the mapped area has NMSE > 0.2. The unnourished time-averaged spatially smoothed mean depth, 〈*d*〉_*smooth*_, is used to fill in small gaps in maps where necessary to allow for the integration over the full domain of interest, to complete the volume estimate calculation.

The volume and beach width (below) estimates are insensitive to the survey resolution and mapping details. Volume estimates were compared using 50-m and 100-m alongshore bins at Cardiff, and using 100-m and 200-m bins at Imperial, Solana and North Torrey Pines (error bars in Figure 9 of ref.^23^). At all sites, on both nourished and unnourished sections, volume estimates with lowered alongshore resolution were not significantly degraded. Volume estimates are also similar using the present constant 5 m cross-shore bin width, and variable cross-shore width bins (each bin instead spans 15 cm vertically on the average profile in ref.^23^). The conceptually simpler evenly spaced cross-shore grid, with cross-shore coordinate origin at the average shoreline position, is used here.

#### Beach widths

Beach width is calculated along each MOP line as the positive slope intersection of the MSL contour with the mapped profile where NMSE < 0.2 (Fig. 4d). In the relatively few cases with multiple MSL intersections, the most offshore MSL position is used, as long as no negative slope intersection is seaward of it. Alongshore-averaged beach widths are provided for each beach (Table 1), region (Table 2) and section (Table 4) when less than 10% of MOP lines were missing estimates.

### Analysis

All code and files used in processing and figure creation are included in this repository folder. All data files created and used in processing are formatted in the Network Common Data Form (NetCDF) and can be read using MATLAB, Python, Fortran, C, C++, Java, and other languages.

## Data Records

The data can be obtained from the Dryad Repository^46^. The files, listed in Table 5, are in NetCDF format^47^, and provide detailed metadata for each variable within the file, using CF conventions 1.6^48^ with Standard Name Table v64.

**Table 5:** Repository files.

Folder	# Data Files	Filenames
water_levels.nc	1	water_levels.nc
Torrey_waves.zip	79	MOP00###_Waves.nc^a^
torrey_survey_info.zip	1	torrey_survey_info.nc
torrey_raw_sand_elevations	255	FILENAMES.nc^b^
torrey_binned_elevations.zip	255	binnedFILENAMES.nc^b^
torrey_mapped_elevations.zip	255	mapFILENAMES.nc^b^
torrey_beach_characteristics.zip	8	torrey_MOP_definitions.nc
		torrey_MOP_definitions.kml
		torrey_regions.nc
		torrey_sections.nc
		torrey_nourishment.nc
		torrey_hard_substrate.nc
		torrey_volumes.nc
		torrey_beach_width.nc
Cardiff-Solana_waves.zip	45	MOP00###_Waves.nc^a^
cardiff-solana_survey_info.zip	1	cardiff_solana_survey_info.nc
cardiff-solana_raw_sand_elevations	188	FILENAMES.nc^b^
cardiff-solana_binned_elevations.zip	188	binnedFILENAMES.nc^b^
cardiff-solana_mapped_elevations.zip	188	mapFILENAMES.nc^b^
cardiff-solana_beach_characteristics.zip	9	cardiff-solana_MOP_definitions.nc
		cardiff-solana_MOP_definitions.kml
		cardiff-solana_regions.nc
		cardiff-solana_sections.nc
		cardiff_nourishment.nc
		solana_nourishment.nc
		cardiff-solana_hard_substrate.nc
		cardiff-solana_volumes.nc
		cardiff-solana_beach_width.nc
Imperial_waves.zip	42	MOP00###_Waves.nc^a^
imperial_survey_info.zip	1	imperial_survey_info.nc
imperial_raw_sand_elevations	95	FILENAMES.nc^b^
imperial_binned_elevations.zip	95	binnedFILENAMES.nc^b^
imperial_mapped_elevations.zip	95	mapFILENAMES.nc^b^
imperial_beach_characteristics.zip	9	imperial_MOP_definitions.nc
		imperial_MOP_definitions.kml
		imperial_regions.nc
		imperial_sections.nc
		imperial_pier_and_jetty_locations.nc
		imperial_nourishment.nc
		imperial_hard_substrate.nc
		imperial_volumes.nc
		imperial_beach_width.nc
analysis	—	analysis_code^c^
		torrey_analysis_intermediate_products^c^
		cardiff-solana_analysis_intermediate_products^c^
		imperial_analysis_intermediate_products^c^
		figures_and_tables^c^

^a^‘###’ corresponds to last 3 digits of MOP site names ‘D0###’.

^b^FILENAMES are formatted as [date, ‘_’, southmostMOP, ‘_’, northmostMOP, ‘_beachSite’, regions, ‘_’, depthZones, ‘_’, vehicles] and when nourishment influence was significant, [‘_nourished’, nourishmentInfluence] is appended.

^c^This is a folder within the analysis folder.

## Technical Validation

### Water levels

All water level sensors were leveled^49^ and data quality controlled^50^. Predictions are generated from harmonic constituents of the record^51^ dating back to 1924^52^.

### Waves

The nearshore wave hindcasts were validated using shallow water wave buoys (20 m depth)^11^. The hourly buoy-driven wave hindcasts show significant skill at most validation sites, but prediction errors for individual swell or sea events can be large. Model skill is high at the sites in north San Diego County, but only fair at Imperial Beach in south San Diego County, owing to a combination of swell energy sensitivity to shadowing by the offshore islands and poorly resolved model bathymetry south of the U.S-Mexico border. Overall, the buoy-driven model hindcasts have relatively low bias such that averaging over space or time is useful for minimizing noise.

### Sand levels

Errors in survey elevation are variable in space and time, and depend on GNSS-platform, bed smoothness, and wave and ocean temperature stratification conditions. Root-mean-square-errors are usually less than 15 cm with the jet ski^53^, and a few cm smaller with the dolly and ATV. Gaps in spatial coverage occurred when low and high tide surveys did not overlap, owing to the nonlinear interaction of sand bars, waves, tides, kelp, divorce, permits, and mechanical failures. Pre- and post- survey control points were used for accuracy verification on each survey, and offsets and antenna heights are included in the raw sand elevation metadata when available. The Online Positioning User Service (OPUS) was used to determine base locations and survey control locations. Inertial measurement units were calibrated on the vehicles and tested. Realtime ocean surface water temperature was recorded during the jet ski surveys to correct for the sonar travel time measurements. Jet ski, dolly, and ATV data are collected over the same transect line with overlap for redundancy and as a check on data quality. Vertical discrepancies are flagged and outliers are removed. The sonar and IMU are oversampled to improve noise rejection. ATV tire pressure is held at 5 PSI and verified prior to each survey. Various jet ski parameters were set at thresholds that maintained high quality (e.g. 30 degree max pitch/roll, maximum Position of Dilution of Precision of 5.0).

## Usage Notes

### Water levels

The sensors at the end of the Scripps pier are less than 40 km away from all monitoring sites and measure the water level above a water column that is approximately 6 m deep. Regional, non-tidal effects (e.g. El Niño) are included in the observed water levels and will be similar between sites and the gauge. However, local effects may vary between the monitoring sites and the gauge observations, particularly eddy activity and wave set down (or some wave setup, if waves are large). Note that when creating shoreline water level estimates, the user must account for wave setup and runup that is not observed in deeper water at the gauge. The tidal-only information provided by the predictions may also vary slightly from the monitoring sites and can be estimated using tidal models (e.g. ADCIRC^54^ or TPXO^55^).

### Waves

On rare occasions, wave model output is degraded due to buoy malfunctions and is flagged using the “waveFlagPrimary” variable. (Good model output has waveFlagPrimary = 1.) Best practices for using the 100 m spaced, 10 m depth MOP wave hindcasts, as boundary conditions for beach change models, are not well established. It is not known if alongshore averaging or smoothing of the 100 m-spaced MOP hindcasts (eg. on typical sea, swell or infragravity wavelength scales) is beneficial for beach change model stability. Space-time wave averaging questions must be explored by investigators based on their specific modeling needs and goals. To reduce small-scale noise in both waves and sand elevation profiles, ref.^13^ used average profiles and waves over several adjacent cross-shore transects. Furthermore, using the fixed shore normal *S*_*xy*_ estimates with 2D beach change models that predict changes in shoreline orientation is internally inconsistent, so additional second-order rotations of the *S*_*xy*_ values (or direct recalculation of *S*_*xy*_ using the *a*_2_ and *b*_2_ Fourier coefficients in compass coordinates) based on modeled shore normal changes, will be required.

### Sand levels

Binned and mapped sand levels are more user-convenient than raw sand elevations for many applications due to the consistent grid locations, but the 5 × 100 m cross-shore x alongshore bin sizes may obscure smaller scale features. Raw data should be used to examine vertical scarps at the seaward face of nourishments, and steep reef and canyon bathymetry. Be cautious with data at depths greater than 8 m below MSL, as ocean temperature stratification can contaminate jet ski soundings. When characterizing unnourished profile behavior in the alongshore uniform sections, alongshore averaging helps to smooth out potentially unresolved features (e.g. beach cusps).

### Complementary datasets

Additional wave data are at http://thredds.cdip.ucsd.edu/thredds/catalog.html.

Additional water level observations are at https://tidesandcurrents.noaa.gov/api/.

Airborne subaerial LIDAR surveys of the southern California coastline (encompassing the survey sites) conducted before and during the monitoring period are available at https://coast.noaa.gov/dataviewer/.

Biannual bathymetric surveys spanning San Diego county, separated on average by a few km, have been acquired by Coastal Frontiers Inc. since 1996 http://www.coastalfrontiers.com.

NCEX bathymetric surveys, that include deeper depths than the surveys presented here, are at http://science.whoi.edu/users/elgar/NCEX/ncex.html.

## ISA-Tab metadata file

Download metadata file

## Data Availability

Code is written in MATLAB (R2018b). Although MATLAB is a proprietary language, the.m files can be read with a text viewer.
